# Comparison of the In Vitro Bioavailability of Selected Minerals from Gluten-Free Breads Enriched with Grains and Synthetic Organic and Non-Organic Compounds

**DOI:** 10.3390/molecules26072085

**Published:** 2021-04-06

**Authors:** Anna Rogaska, Julita Reguła, Joanna Suliburska, Zbigniew Krejpcio

**Affiliations:** Department of Human Nutrition and Dietetics, Poznan University of Life Sciences, Wojska Polskiego St. 31, 60-624 Poznan, Poland; anna.rogaska89@gmail.com (A.R.); joanna.suliburska@up.poznan.pl (J.S.); zbigniew.krejpcio@up.poznan.pl (Z.K.)

**Keywords:** in vitro, bioavailability, iron, copper, zinc, magnesium, calcium, gluten-free breads

## Abstract

Introduction: Despite the constant efforts of scientists to improve the texture, sensory properties, and nutritional value of gluten-free bread, obtaining high bioavailability of minerals is still a huge challenge. Gluten-free bakery products are characterized by a low bioavailability of minerals. The aim of this study was to design gluten-free bread with high bioavailability of minerals commonly found in deficiencies in people struggling with gluten intolerance. Material and methods: The material consisted of gluten-free breads designed to obtain the highest possible content of minerals in the bread while maintaining a good structure and taste. Results: Higher contents of all the analyzed minerals were obtained in breads with natural and synthetic additives, both in rice and buckwheat bread, compared to basic bread. There was also a higher content of the analyzed minerals in buckwheat bread in comparison to rice bread for each type of additive. Higher bioavailability of iron, copper, calcium, and magnesium was noted in rice bread, while the bioavailability of zinc was higher in buckwheat bread. Conclusion: The additives used increased the bioavailability of the analyzed minerals from the gluten-free breads. The use of various variants of flour (rice, buckwheat) influenced the bioavailability of iron, zinc, copper, calcium, and magnesium. The release of minerals from gluten-free bread depends on the element and added components (seeds or synthetic additives).

## 1. Introduction

In light of the applicable law, gluten-free bread is a food for special nutritional uses. This means that it is dedicated to people struggling with special nutritional needs, including gluten intolerance [1]. In people with gluten intolerance, after consuming gluten, and more specifically its gliadin fraction, intestinal villi are damaged and atrophied, which, apart from digestive system ailments, leads to impaired absorption of nutrients. Gluten-free bread is baked using gluten-free flour [2]. Despite the constant efforts of scientists to improve the texture, sensory properties, and nutritional value of gluten-free bread, obtaining high bioavailability of minerals is still a huge challenge [2]. In people struggling with gluten intolerance, degradation of the intestinal villi occurs, which results in a low digestibility of food ingredients [3]. In addition, gluten-free bakery products available on the market are characterized by a low bioavailability of minerals, which increases this nutritional deficiency [4]. Research shows that the bioavailability of Ca from gluten-free bread reaches 9%, Mg 21%, Fe 38%, Zn 50%, and Cu 62% [5]. The bioavailability of minerals—the degree of their use in the human body—depends on many nutritional and non-nutritional factors. The most important of these include age, sex, physiological state, nutritional status, source of the mineral, form of occurrence, method of technological processing, and interactions among minerals [5,6]. The bioavailability of minerals decreases with age, depends on the level of sex hormones in the body, is higher for pregnant women, and lower with a high saturation of the given minerals [7]. In the case of iron, higher bioavailability is observed in fermented products in the presence of hydrochloric acid, vitamin C, lactic and tartaric acids, and lactoferrin. On the other hand, it is lower in the presence of phytic acid and its salts, fiber, Cu, Ca, Zn, Mn, Pb, Cd, and vitamin E, as well as in people who smoke and abuse alcohol, coffee, and tea. In the heme form, 20–30% of iron is absorbed, while in the non-heme form, 1–5% [8]. The bioavailability of zinc is 20–40%, which increases in the presence of carbohydrates, citric acid, animal protein, lactose, vitamins B6, D, A, E, and C as well as hydrochloric acid. Low bioavailability occurs in the presence of phytic acid, fiber, oxalic acid, tannin, non-heme iron, Ca, Cu, Cd, and alcohol [7]. For copper, higher bioavailability is noted in the presence of animal protein and low pH, and lower in the presence of Fe, Zn, Mb, Cd, phytates, sulfur compounds or antacids. On average, 35–40% of copper is absorbed [8]. The bioavailability of calcium is 30–40% on average, which increases in the presence of basic amino acids, lactose, bile salts, inulin, vitamin D, protein, lactic acid bacteria, short-chain fatty acids, phosvitin, and casein. It decreases at high pH and in the presence of fiber, fat, phytates, oxalates, phosphorus, alcohol, Fe, Mg, Mn, Zn, and Cu [9]. For magnesium, the bioavailability is about 50%, and higher bioavailability is obtained in the presence of protein, lactose, unsaturated fatty acids, and vitamins B6 and D. It is lower in the presence of saturated fats, cellulose, phytates, tannins, oxalates, Ca, P, and fiber. The best absorbable forms are citrate, which can, however, have a laxative effect, and ascorbate and aspartate, which, when not bound to other amino acids, can cause neurotoxicity. Gluconate and ornithate are also often found in food, but their absorption reaches a maximum of 50% [7]. Considering the multitude of dependencies affecting the degree of absorption of minerals, the research carried out to improve it seems to be very important. Therefore, the aim was to design gluten-free bread with high bioavailability of minerals commonly found in deficiencies in people struggling with gluten intolerance.

## 2. Results

Table 1 presents the content of the selected minerals in the designed gluten-free breads. Statistically higher contents of all the analyzed minerals were obtained in breads with natural and synthetic additives, both in rice and buckwheat bread, compared to basic bread without additives. The exception was the zinc content in breads, where the highest content was recorded for basic bread without additives; in the case of rice bread, the amounts were high in both basic bread and bread with seeds, compared to bread with synthetic additives. There was also a significantly higher content of the analyzed minerals in buckwheat bread in comparison to rice bread for each type of additive. Rice breads showed a significantly higher content of iron, zinc, and copper for breads with an organic additive compared to non-organic additives. In buckwheat bread, a similar relationship was noted for the content of iron and calcium.

Table 2 shows the nutritional value of the designed gluten-free breads. Higher water and carbohydrate content was observed in rice and buckwheat bread without additives, and higher energy, fat, protein, and fiber content in breads with seeds. Taking into account the type of bread, a significantly higher difference in dietary fiber was demonstrated for both basic buckwheat breads without additives and for bread with added seeds, as well as a significantly higher carbohydrate content for basic rice bread and protein for basic buckwheat bread.

**Figure 1:**
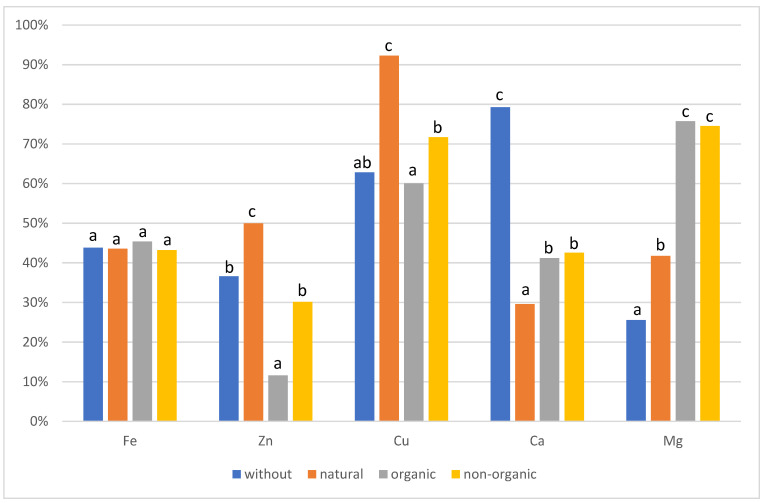
Bioavailability in rice bread, a, b, c statistical differences between additives (seeds, supplements).

**Figure 2:**
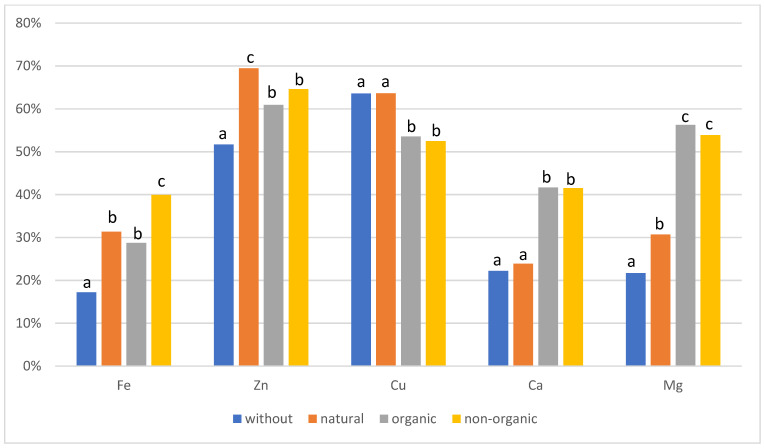
Bioavailability in buckwheat bread, a, b, c statistical differences between additives (seeds, supplements).

The results of the bioavailability of the selected minerals from rice and buckwheat bread are presented in Figure 1 and Figure 2. After in vitro digestion, no significant difference was found in the bioavailability of iron in rice breads. The obtained bioavailability of this component at the level of 43–46% is high. The highest bioavailability of zinc and copper for rice breads was demonstrated for those with a natural additive (50% and 92.2%, respectively), and the lowest for those with an organic additive (11.6% and 60.1%, respectively). Significant differences in the bioavailability of these components were also observed depending on the type of synthetic additive in favor of non-organic compounds. The bioavailability of calcium in rice bread was the highest for the bread without additives (79.2%), and the lowest for bread with a natural additive (29.6%). There were no differences between the synthetic additives. For magnesium in rice breads, the highest bioavailability was obtained in bread with synthetic, organic, and non-organic additives (75.7%). On the other hand, the lowest was found in bread without additives (25.6%).

For buckwheat bread, the highest iron bioavailability was obtained in bread with non-organic additives (39.9%), and the lowest for bread without additives (17.1%). For zinc, the highest bioavailability in buckwheat bread was obtained from bread with a natural additive (69.5%), and the lowest from bread without additives (51.7%). There was no difference between the synthetic additives. For copper, the highest bioavailability was obtained from bread without additives and with a natural additive (63.6%), and the lowest from bread with an organic additive (52.4%). There was no difference between the synthetic additives. For calcium, the highest bioavailability was demonstrated in bread with synthetic, organic, and non-organic additives (41.6%), and the lowest for bread without additives (22.2%). In the case of magnesium, the highest bioavailability was obtained from bread with the addition of organic compounds (56.2%), and the lowest from bread without additives (21.7%). There was no difference between the synthetic additives.

**Figure 3:**
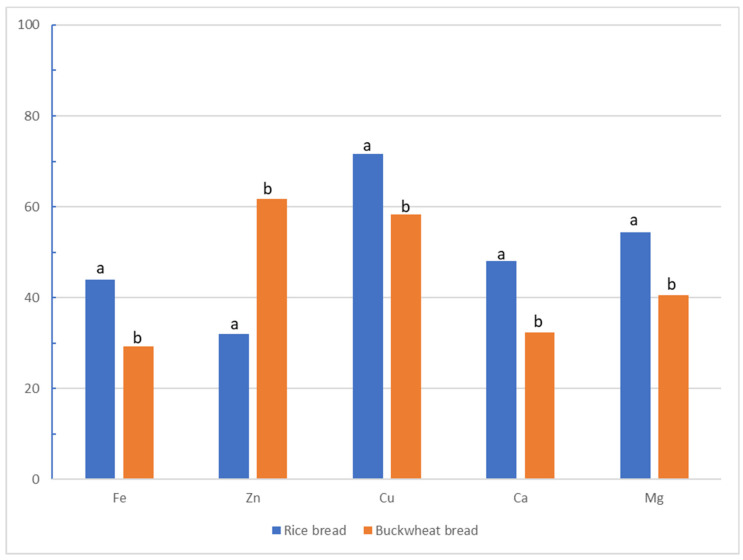
Effects of the type of bread (rice, buckwheat) on the bioavailability of minerals (ANOVA main effects) (%), a, b statistical differences between additives (seeds, supplements).

**Figure 4:**
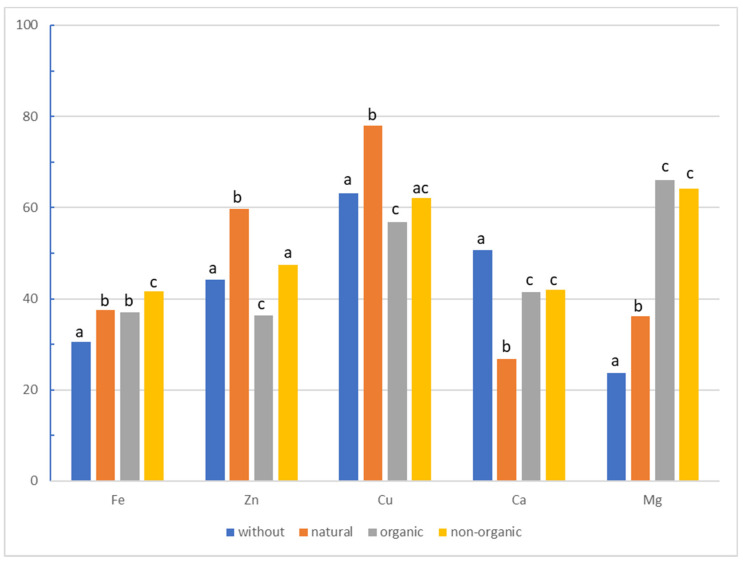
Effects of the type of additive (no additive, seeds, synthetic organic and non-organic compounds) on the bioavailability of minerals (ANOVA main effects) (%), a, b, c statistical differences between additives (seeds, supplements).

Figure 3 and Figure 4 show the results of the main effects of the type of bread and additive on the bioavailability of selected minerals from gluten-free bread. Higher bioavailability of iron, copper, calcium, and magnesium was noted in rice bread, while the bioavailability of zinc was twice as high in buckwheat than in rice bread. The highest bioavailability of iron was obtained from bread with the addition of non-organic compounds (41.5%), and the lowest from bread without additives (30.5%). Additionally, a significant difference was demonstrated between the addition of organic and non-organic compounds. In the case of zinc and copper, the highest bioavailability was obtained from breads with the addition of organic compounds (59.7%, 77.9%, respectively), and the lowest was obtained from breads with the addition of organic compounds (36.3%, 56.8%, respectively). Significant differences between organic and non-organic additives were again demonstrated. For calcium, the highest bioavailability was obtained from bread without additives (50.7%), and the lowest from bread with a natural additive (26.7%). There were no significant differences between organic and non-organic additives. For magnesium, the highest bioavailability was recorded for bread with an organic additive (65.9%), and the lowest for bread without additives (23.6%). Again, no differences were observed between organic and non-organic compounds.

## 3. Discussion

Obtaining high bioavailability of minerals in gluten-free bread has been challenging scientists in the field of human nutrition, dietetics, and food technology for several years [2,10]. The low absorption capacity in people struggling with gluten intolerance and the low content of deficient minerals in breads available on the market give bread producers a chance to create a niche product [11]. However, trying to produce gluten-free bread with high bioavailability of minerals is extremely difficult due to a number of limitations affecting the nutritional value of the product and its organoleptic characteristics. The competitiveness of minerals does not allow for the addition of lots of organic and non-organic compounds to bread [12]. On the other hand, the limiting effect of dietary fiber does not allow a very high proportion of natural ingredients to be used in the designed bread. Finding the golden mean is extremely difficult and requires a lot of research [5]. In people struggling with gluten intolerance, significant deficiencies of minerals occur, leading to further disease entities such as iron deficiency anemia, osteoporosis and osteomalacia, and malnutrition, which has also mobilized scientists to conduct more extensive research in this field [13]. Attempts to obtain a high content of minerals in gluten-free bread are associated with an increasing range of additives being used. The bread is enriched with various natural additives in the form of seeds (flax, sunflower, pumpkin, nuts) [2,4,6], inulin [13,14], and even mushrooms [15,16]. Oilseeds such as flax, sunflower, and pumpkin seeds, and higher fungi such as *Pleurotus ostreatus* and shiitake *Lentinula edodes* are characterized by high nutritional values, antioxidant activity, and a general content of polyphenols [15,16,17,18]. They are a natural, rich source of vegetable protein, polyunsaturated fatty acids, fiber, minerals, vitamins, and phytoestrogens. In addition, oil seeds have a positive effect on the structure, porosity, and moisture of the bread crumb, which is particularly important when obtaining gluten-free products whose structure is weaker than that of traditional bread, due to the lack of gluten [19]. Comparing the obtained results to the research carried out by Suliburska et al., the obtained content of Ca, Mg, Fe, and Cu was 2–3 times higher, and the content of Zn was 2 times lower in gluten-free bread. Higher bioavailability of Ca, Mg, and Cu, comparable bioavailability of Fe, and slightly lower bioavailability of Zn were also obtained. The lower content and bioavailability of zinc was probably related to the competitiveness of other minerals present in the higher content [5]. Krupa-Kozak et al. added buckwheat flour to gluten-free bread in order to enrich it with minerals, but the values obtained by them are twice lower than in the present study [20]. In addition to enriching bread with natural additives, synthetic organic and non-organic substances, which are the carriers of minerals such as lactates, sulfates, gluconates, and carbonates [19,21,22], are also used to enrich it. The use of iron pyrophosphate and iron glycinate increases the bioavailability of iron and does not cause significant sensory changes in the product [10,23]. The addition of iron sulphate in an amount over 5 g changes the palatability of the bread, which is related to its lower acceptance. However, it is both popular and the cheapest iron compound suitable for bread fortification [10,24]. The addition of inulin and oligosaccharides can significantly affect the bioavailability of calcium by producing lactic acid, which lowers the pH of the gastrointestinal tract, promoting the absorption of more minerals [12,14]. Research indicates that the bioavailability of calcium may also be affected by the form of calcium occurrence. Candia et al. have shown that calcium citrate can inhibit iron absorption in fasting people [21]. The most common calcium compounds added to foods are carbonate and citrate [19,22]. The addition of calcium carbonate in the amount of 3 g/kg equalizes the content of this component with the gluten equivalent. On the other hand, calcium citrate is characterized by a higher bioavailability than calcium carbonate, further reducing the amount of hydrochloric acid secreted in the stomach [13,25]. Calcium gluconate and calcium lactate are forms with a lower concentration of calcium, though they are most often used to supplement bread [19]. Bread is one of the best-tested food products in terms of organoleptic, structural, and rheological characteristics as well as nutritional value. Due to the commonness of its consumption, it is a good carrier of many substances [18]. Due to the rather small amount of studies, it is difficult to compare the results of the bioavailability of minerals from gluten-free products [5]. However, there is still a lack of research answering the question of what to do to improve the bioavailability of minerals from gluten-free bread while maintaining the appropriate structural and sensory properties [26].

## 4. Materials and Methods

### 4.1. Bread Recipes

The research material consisted of gluten-free breads designed to obtain the highest possible content of deficient minerals in the bread while maintaining a good structure and taste. The bread was produced in two variants: light-rice and dark-buckwheat, with the same number of additives.

Table 3 shows the content of raw materials used for baking individual types of bread. In rice and buckwheat bread, only the basic raw materials needed to bake bread were used. Poppy seeds, flax, hazelnuts, amaranth, pumpkin seeds, sunflower seeds, and egg yolk were added to the bread with seeds replacing some of the basic flours. Breads with added organic and non-organic compounds were designed so that the content of minerals was similar to the corresponding bread with natural additives.

Table 4 shows the amounts of organic and non-organic compounds used to enrich the designed gluten-free bread with selected minerals. The present organic and non-organic compounds were added to the flour in powder form.

### 4.2. Baking Bread

The breads were baked using the two-phase method. The dough was raised in a heat chamber for 40 min at 35 °C, then baked in an oven for 23 min at 195 °C.

### 4.3. Assessment of Mineral Content

After grinding slices of bread (with both crumb and crust), all laboratory samples were transferred at an amount of 2 g into quartz crucibles, each in 3 repetitions. Their water content was determined using the drying method, and the dried samples were incinerated in a muffle furnace (Nabertherm P330, GmbH, Lilienthal, Germany) at 250–450 °C. The resulting ash was dissolved in 1N HNO_3_ (Merck, Darmstadt, Germany) and transferred quantitatively to volumetric flasks. In samples (solution), after dilution with 1N HNO_3_ solution (additionally for Ca and Mg with LaCl_3_ solution), the contents of Ca, Mg, Fe, Zn, and Cu were determined using flame atomic absorption spectrometry (ASA) using an AAS-3 spectrometer (Zeiss, Jena, Germany). The accuracy of the method was determined against a certified reference material (Brown Bread BCR191, Sigma-Aldrich, Darmstadt, Germany), and the percentage recovery for Ca, Mg, Fe, Zn, and Cu, respectively, was 94%, 98%, 91%, 93%, and 103% [2].

### 4.4. Assessment of Mineral Bioavailability In Vitro

The samples were subjected to enzymatic digestion in vitro to determine the potential relative bioavailability of Ca, Mg, Fe, Zn, and Cu [10]. From each material, 2 g of the sample was weighed in a conical flask, and 20 mL of deionized water was added. Then, the pH was adjusted to 2.0 with 0.1N HCl solution and treated with pepsin (0.5 mL/100 mL homogenate). The samples were shaken for 2 h in a thermostatic water bath (temperature 37 °C), controlling the pH. The pH was then raised to 6.8–7.0, and it was treated with pancreatin (10 mL/40 mL of homogenate) and shaken again in a water bath for 4 h. After the digestion process was completed, the samples were centrifuged for 10 min at a speed of 3800 rpm. Then, 10 cm^3^ of the supernatant was transferred into Teflon vessels, and 5 cm^3^ of concentrated nitric acid (Merck) was added, and then mineralized in a MARS-5 microwave oven (CEM Corp.). The concentration of the elements Ca, Mg, Fe, Zn, and Cu was determined using spectrometry atomic absorption (AAS-3 spectrometer, Zeiss). The measurements were repeated three times. The accuracy of the method was determined against a certified reference material (Brown Bread BCR191, Sigma-Aldrich), and the percentage recovery for Ca, Mg, Fe, Zn, and Cu, respectively was 105.6%, 95.4%, 94.0%, 91.8%, and 98.8% [2].

The relative bioavailability potential is expressed as a percentage of the amount of a mineral released during enzymatic digestion per unit weight of product to the total amount of mineral contained in a unit weight of product.

The mineral contents in samples were measured at wavelengths of 248.3 nm for Fe, 213.9 nm for Zn, and 324.8 nm for Cu. Deionized water and acid-washed glassware were used in this study.

### 4.5. Nutrition Value

The amounts of dry matter, protein, and crude fat were determined in the breads using standard analytical methods [27]. Dry matter was determined by drying 1 g of sample in an oven at 105 °C for 12 h and weighing. Ash content was determined by incineration at 550 °C for 24 h. Total fat was determined via the Soxhlet extraction method. Protein content was determined via the Kjeldahl total nitrogen method. Dietary fiber content was determined by the Aspa enzymatic–gravimetric method. Carbohydrates constituted the difference of 100 minus the sum of water, ash, protein, and fat contents. To establish the availability of energy from bread and the amount of metabolic energy uptake, gross energy in the bread was determined using a KL-10 adiabatic oxygen bomb calorimeter (SP Precyzja, Bydgoszcz, Poland), according to Polish Standards PN-ISO 1928:2002 [28]. Metabolic energy of the breads was calculated from the equation: Metabolic energy = (0.95 × WED) − (0.75 × N) where WED is the gross energy value of the bread (MJ) and N is the amount of nitrogen (g/kg of bread) [29].

### 4.6. Statistical Analysis

In order to determine the effect of the additive and type of bread on in vitro bioavailability, the analysis of variance was applied, while the significance of intergroup differences was assessed using one-way or two-way ANOVA with post-hoc Tukey’s test. All differences were considered to be statistically significant at a 5% probability level. Data were analyzed with Statistica 13.0 software.

## 5. Conclusions

The natural additives used, as well as the organic and non-organic compounds, increased the bioavailability of the analyzed minerals from the newly designed gluten-free breads. The use of various variants of flour (rice, buckwheat) influenced the bioavailability of iron, zinc, copper, calcium, and magnesium. Higher bioavailability of iron, copper, calcium, and magnesium was found in rice bread, and of zinc in buckwheat.

The addition of seeds to the bread increased the bioavailability of zinc (in buckwheat bread) and copper (in rice bread) and decreased the bioavailability of calcium.

The addition of synthetic compounds, both organic and non-organic, to the breads significantly increased the bioavailability of magnesium and in the case of buckwheat bread, also calcium. The release of minerals from gluten-free bread depends on the element and added components (seeds or synthetic additives). The use of a seed supplement is recommended to increase the absorption of zinc, copper, and calcium. The use of synthetic additives increases the bioavailability of magnesium.

## Figures and Tables

**Table 1:** Content of selected minerals in breads.

**Minerals (mg/100 g d.m.)**	**No Addition**	**Seeds**	**Organic**	**Non-Organic**
	**R**	**RS**	**sRo**	**sRn**
Fe	1.19 ± 0.04 ^aA^	2.28 ± 0.01 ^dA^	2.2 ± 0 ^cA^	1.97 ± 0.02 ^bA^
Zn	1.27 ± 0.01 ^cA^	1.22 ± 0.04 ^cA^	1.06 ± 0.04 ^aA^	1.17 ± 0.02 ^bA^
Cu	0.214 ± 0.016 ^aA^	0.282 ± 0.013 ^bA^	0.261 ± 0.014 ^bA^	0.228 ± 0.005 ^aA^
Ca	26.2 ± 0.8 ^aA^	131.3 ± 5.8 ^bA^	133.8 ± 4.4 ^bA^	129.5 ± 4.8 ^bA^
Mg	18.9 ± 0.4 ^aA^	89 ± 1.5 ^cA^	80.1 ± 0.4 ^bA^	79.6 ± 0.8 ^bA^
**Minerals (mg/100 g d.m.)**	**No Addition**	**Seeds**	**Organic**	**Non-Organic**
	**B**	**BS**	**sBo**	**sBn**
Fe	2.06 ± 0.13 ^aB^	3.17 ± 0.02 ^cB^	2.73 ± 0.01	2.23 ± 0.03 ^aB^
Zn	1.88 ± 0.06 ^bB^	1.33 ± 0.04 ^aB^	1.47 ± 0.01	1.44 ± 0.06 ^aB^
Cu	0.297 ± 0.011 ^aB^	0.495 ± 0.004 ^bB^	0.47 ± 0.022	0.458 ± 0.001 ^bB^
Ca	33 ± 0.6 ^aB^	143.1 ± 2.1 ^dB^	146.9 ± 1.1	136.9 ± 0.5 ^bB^
Mg	86.6 ± 1.6 ^aB^	128.6 ± 5.7 ^bB^	126 ± 4.5	122.4 ± 1.5 ^bB^

R—basic rice bread, B—basic buckwheat bread, RS—rice bread with seeds, BS—buckwheat bread with seeds, sRo—rice bread with organic additives, sBo—buckwheat bread with organic additives, sRn—rice bread with non-organic additives, sBn—buckwheat bread with non-organic additives. a, b, c and d indicate statistical differences between additives (seeds, supplements). A, B, indicate statistical differences between types of bread (rice, buckwheat).

**Table 2:** The energy value and the content of macronutrients in the main breads (rice and buckwheat) [2] and breads with seeds.

**Components**	**No Addition**	**Seeds**
	**R**	**RS**
Energy (kcal) (MJ)	236.3 ± 3.5 ^aA^ 0.987 ± 0.014	294.1 ± 4.01 ^bA^ 0.965 ± 0.01
Water (g/100 g)	42.2 ± 0.92 ^bA^	38.54 ± 1.13 ^aA^
Fat (g/100 g)	1.05 ± 0.05 ^aA^	10.75 ± 0.99 ^bA^
Protein (g/100 g)	4.21 ± 0.08 ^aA^	10.14 ± 1.42 ^bA^
Carbohydrates (g/100 g)	52.4 ± 0.97 ^bB^	39.3 ± 0.99 ^aA^
Fiber (g/100 g)	3.78 ± 0.12 ^aA^	16.25 ± 0.35 ^bA^
**Components**	**No addition**	**Seeds**
	**B**	**BS**
Energy (kcal) (MJ)	236.9 ± 1.9 ^aA^ 0.99 ± 0.007	298.97 ± 4.11 ^bA^ 1.251 ± 0.01
Water (g/100 g)	43.2 ± 0.38 ^bA^	38.78 ± 1.12 ^aA^
Fat (g/100 g)	1.95 ± 0.07 ^aA^	12.29 ± 1.23 ^bA^
Protein (g/100 g)	6.78 ± 0.09 ^aB^	11.33 ±1.34 ^bA^
Carbohydrates (g/100 g)	48.0 ± 0.2 ^bA^	35.76 ± 1.11 ^aA^
Fiber (g/100 g)	4.97 ± 0.14 ^aB^	19.26 ± 0.85 ^bB^

R—basic rice bread, B- basic buckwheat bread, RS–rice bread with seeds, BS—buckwheat bread with seeds. a, b, indicate statistical differences between additives (seeds, supplements). A, B indicate statistical differences between type of breads (rice, buckwheat).

**Table 3:** Composition of breads (g/100 g).

Component	R	B	RS	BS
Buckwheat flour	0	33	0	27
Rice flour	33	0	27	0
Corn starch	17	17	13	13
Potato starch	33	33	21	21
Pectin	4	4	4	4
Yeast	4	4	4	4
Sugar	5	5	3	3
Salt	1	1	1	1
Rapeseed oil	3	3	2	2
Poppy seeds	0	0	5	5
Amaranth flour	0	0	3	3
Golden flax	0	0	4	4
Sunflower seeds	0	0	4	4
Pumpkin seeds	0	0	4	4
Hazelnuts	0	0	3	3
Egg yolk	0	0	2	2

R—basic rice bread, B—basic buckwheat bread, RS—rice bread with seeds, BS—buckwheat bread with seeds.

**Table 4:** The amounts of organic and non-organic compounds used as the source of selected minerals added to the breads (mg/100 g d.m.).

Minerals	Type of Bread	Organic Compound		Non-Organic Compound	
Fe	R	Iron gluconate (II)	50.4	Iron (II) sulfate (VI), heptahydrate	31.4
	B		50.8		31.6
Cu	R	Cooper D-gluconate (II)	2.80	Cooper (II) sulfate (VI)	0.99
	B		8.05		2.83
Ca	R	Calcium lactate, pentahydrate	4667	Calcium carbonate	1515
	B		4811		1562
Mg	R	Magnesium l-lactate, hydrate	3371	Magnesium carbonate	1403
	B		1986		827

R–basic rice bread, B–basic buckwheat bread.

## Data Availability

The data presented in this study are available on request from the corresponding author.

## Sample Availability

Samples of the compounds are not available from the authors.
